# Apparent Polyploidization after Gamma Irradiation: Pitfalls in the Use of Quantitative Polymerase Chain Reaction (qPCR) for the Estimation of Mitochondrial and Nuclear DNA Gene Copy Numbers

**DOI:** 10.3390/ijms140611544

**Published:** 2013-05-30

**Authors:** Winnie W. Y. Kam, Vanessa Lake, Connie Banos, Justin Davies, Richard Banati

**Affiliations:** 1 Australian Nuclear Science and Technology Organisation, Lucas Heights, Sydney, New South Wales 2234, Australia; E-Mails: val@ansto.gov.au (V.L.); cbx@ansto.gov.au (C.B.); jbd@ansto.gov.au (J.D.); rib@ansto.gov.au (R.B.); 2 Medical Radiation Sciences, Faculty of Health Sciences, University of Sydney, Cumberland, Sydney, New South Wales 2141, Australia; 3 School of Physics, University of Sydney, Camperdown, Sydney, New South Wales 2006, Australia; 4 National Imaging Facility at Brain and Mind Research Institute (BMRI), University of Sydney, Camperdown, Sydney, New South Wales 2050, Australia

**Keywords:** gamma radiation, temperature, mitochondria, nucleus, DNA copy number, polyploidization

## Abstract

Quantitative polymerase chain reaction (qPCR) has been widely used to quantify changes in gene copy numbers after radiation exposure. Here, we show that gamma irradiation ranging from 10 to 100 Gy of cells and cell-free DNA samples significantly affects the measured qPCR yield, due to radiation-induced fragmentation of the DNA template and, therefore, introduces errors into the estimation of gene copy numbers. The radiation-induced DNA fragmentation and, thus, measured qPCR yield varies with temperature not only in living cells, but also in isolated DNA irradiated under cell-free conditions. In summary, the variability in measured qPCR yield from irradiated samples introduces a significant error into the estimation of both mitochondrial and nuclear gene copy numbers and may give spurious evidence for polyploidization.

## 1. Introduction

Mitochondria, organelles with a broad range of functions from the provision of cellular energy to signalling, cell cycle and growth, have their own genome consisting of multiple copies of circular DNA [1]. Using quantitative polymerase chain reaction (qPCR), increases in the amount mitochondrial DNA copies have been reported after ionizing irradiation *in vivo* [2–6] and *in vitro* [7–9]. These observations are being interpreted as mitochondrial polyploidization [4], an apparent compensatory effect in response to stress-induced mitochondrial DNA depletion in order to maintain cellular energy status and survival [2,4,10].

The electron transport chain is a system that resides within mitochondria for respiration therefore, generation of chemical energy in the form of adenosine triphosphate. It is composed of four enzymatic complexes, and, together with ATP synthase (Complex V), there are 13 subunits encoded by mitochondrial DNA as well as 77 subunits encoded by nuclear DNA [11]. Using specific PCR primers, a gene segment encoding either the mitochondrial or nuclear subunit of the same complex can be selectively amplified from a total DNA template (which is a pool of mitochondrial and nuclear DNAs). The qPCR yield of the mitochondrial or nuclear gene reflects the initial amount of DNA template in a sample and, thus, gives information about the gene copy numbers of the mitochondria or nucleus, respectively.

The present study describes an important limitation in the use of qPCR for the quantification of both nuclear and mitochondrial gene copy numbers after gamma irradiation, due to radiation-induced fragmentation of the DNA templates.

## 2. Results and Discussion

The standard curve method generates absolute concentrations by comparing unknown samples to known, which are usually cDNA or TA cloned PCR product. These external standards are intrinsically different from the experimental PCR templates—as in this study, the total DNA from a cell. The amplifiability (the primer’s ability to hybridize to and initiate amplification from the template) of the same gene between the experimental samples and external standard should differ, likely due to the difference in template complexity.

We, therefore, quantified our qPCR data using the ΔCq method, which directly compares the yield of the same gene of the same type of material (total DNA from JURKAT cells) before and after irradiation, *i.e.*, ΔCq = Cq (un-irradiated sample) − Cq (irradiated sample). Such a quantification approach allows a clear delineation on the effect of radiation on the change in PCR product yield. This quantification method also avoids the “dilution bias” described by Malik *et al.* [12,13].

The amount of template doubles after each cycle during the linear phase of a PCR; thus, the fold change in the amount of PCR product of the irradiated relative to that of an un-irradiated sample can be quantified as follows: 2 ^Cq (un-irradiated sample) − Cq (irradiated sample)^[14,15]. The final quantified results are presented as a fold change in the amount of PCR product after irradiation; therefore, the observed qPCR artefact (see below) can be clearly illustrated using line plots.

### 2.1. qPCR Yield from Cells after Radiation Exposure

**Figure 1:**
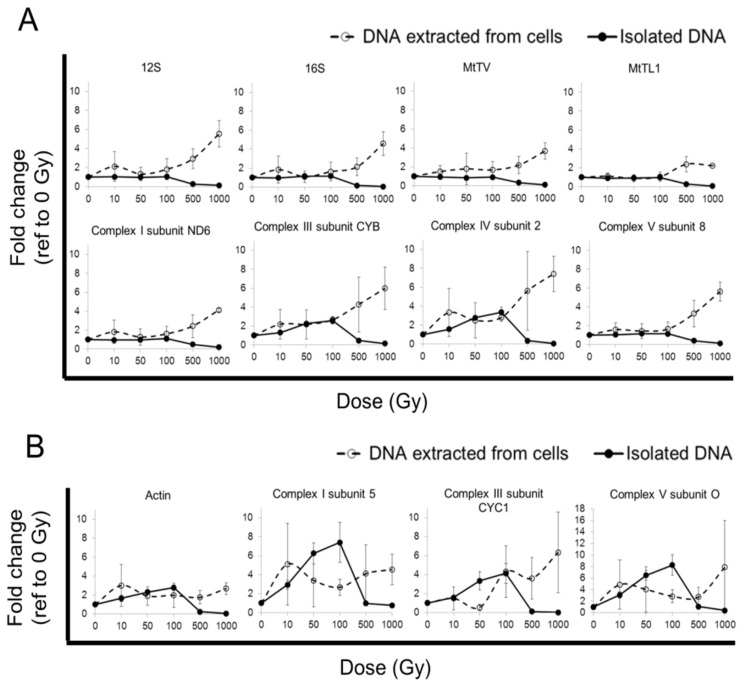
Gene copy number quantification in cells or isolated (cell-free) total DNA after gamma irradiation at 21 °C. JURKAT cells (dashed line) or isolated total DNA (solid line) (*N* = 3) were exposed to 0 (control), 10, 50, 100, 500 and 1000 Gy of gamma radiation at room temperature (~21 °C). DNA samples were subjected to qPCR and quantified with reference to the un-irradiated sample: 2 ^Cq (un-irradiated sample) − Cq (irradiated sample)^. The fold change in gene copy number of the tested mitochondrial (**A**) and nuclear (**B**) genes is shown. The data show the variation of fold change with radiation dose in the genes amplified from DNA extracted from irradiated cells or directly irradiated cell-free DNA.

When cells were irradiated at room temperature, mitochondrial gene copy numbers, as measured by qPCR yield, appeared to increase with dose (Figure 1A; dashed lines), suggesting an increase in total mitochondrial DNA. The apparent radiation-induced increase in mitochondrial DNA affects mitochondrial genes differentially, *i.e.*, the increase in qPCR yield of different genes of the same mitochondrial genome varied strongly (Figure 1A; dashed lines).

The measured qPCR yields for nuclear genes, too, increased acutely after irradiation, suggestive of an apparent increase in nuclear gene copy numbers (Figure 1B; dashed lines).

### 2.2. qPCR Yield from Isolated DNA after Radiation Exposure

In order to examine the aforementioned apparent increases in gene copy numbers independently of any cellular biological processes, *i.e.*, DNA replication, DNA isolated from JURKAT cells was irradiated with the same dose and under the same condition as the cells *in vitro*. The irradiated cell-free DNA was subsequently used for qPCR. An inconsistent increase in qPCR yields, interpreted as an increase in gene copy numbers, was seen across the tested mitochondrial (Figure 1A; solid lines), as well as nuclear genes (Figure 1B; solid lines).

For example, a ~100% and 250% increase in Complex III subunit CYB and Complex IV subunit 2 genes, respectively, could be detected at 100 Gy. In contrast, the rest of the tested mitochondrial genes at the same dose of 100 Gy showed only a very small increase (~3% to 16%). qPCR yield is known to inversely relate to the severity of DNA damage [16–19]; thus, decreases in yield were seen at 500 to 1000 Gy (Figure 1A; solid lines).

Similarly, the nuclear genes amplified from cell-free DNA also showed apparent and variable increases in gene copy numbers ranging from ~100% to 700%, especially at 100 Gy. As seen previously in the mitochondrial genes, only a minor amount of nuclear genes could be amplified at 500 to 1000 Gy (Figure 1B, solid lines).

### 2.3. Gel Analysis of DNA Template Fragmentation after Radiation Exposure

The integrity of the DNA templates used in the above qPCR assays was analysed by 1.5% agarose gel electrophoresis. This gel concentration was chosen to detect small size DNA fragments, as these increase markedly if a substantial amount of fragmentation has occurred.

**Figure 2:**
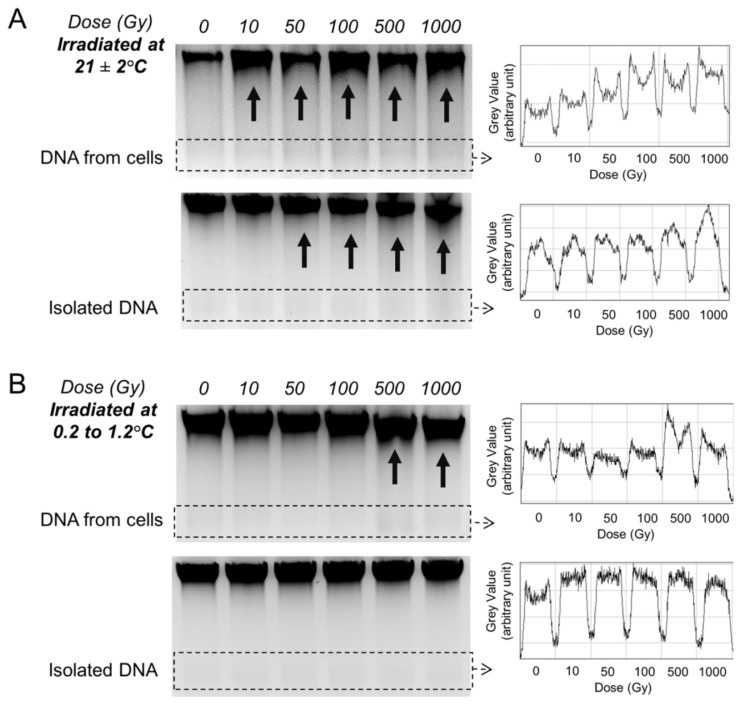
Gel electrophoresis of the DNA templates. (**A**) Equal amounts of DNA extracted from JURKAT cells (260 ng/lane, top image) or isolated total DNA (1 μg/lane, bottom image) irradiated at room temperature were loaded into a 1.5% agarose gel for electrophoresis. Similarly, the same procedure was performed using (**B**) DNA extracted from JURKAT cells (1 μg/lane, top image) or isolated DNA (1 μg/lane, bottom image) irradiated at low temperature. The level of DNA fragmentation of each sample (boxed area) is demonstrated by the line profile plot presented next to the gel image. Arrows indicate DNA smearing or size reduction of the major band. The data show a radiation dose-dependent increase in DNA fragmentation that can be reduced by lowering the irradiation temperature from ~21 °C to 0.2–1.2 °C.

For the DNA extracted from cells irradiated at room temperature, smearing of the DNA (Figure 2A, top image, arrows) and radiation dose-dependent formation of small fragments was observed (Figure 2A, top image, boxed region and profile plot).

For cell-free DNA irradiated at room temperature, too, an increase in small fragments was seen (Figure 2A; bottom image, profile plot). A gradual size reduction of the major band together with smearing was observed, most notably at dose levels of 50 Gy and above (Figure 2A; bottom image, arrows).

### 2.4. qPCR Yield from Cells after Radiation Exposure at Low Temperature

The radio-protective effect of low temperature has been well described *in vitro* [20–25] and *in vivo* [26]. We, therefore, attempted to reduce the level of radiation damage by irradiating cells and cell-free DNA on ice (0.2 to 1.2 °C) and to investigate the subsequent change in qPCR yield.

**Figure 3:**
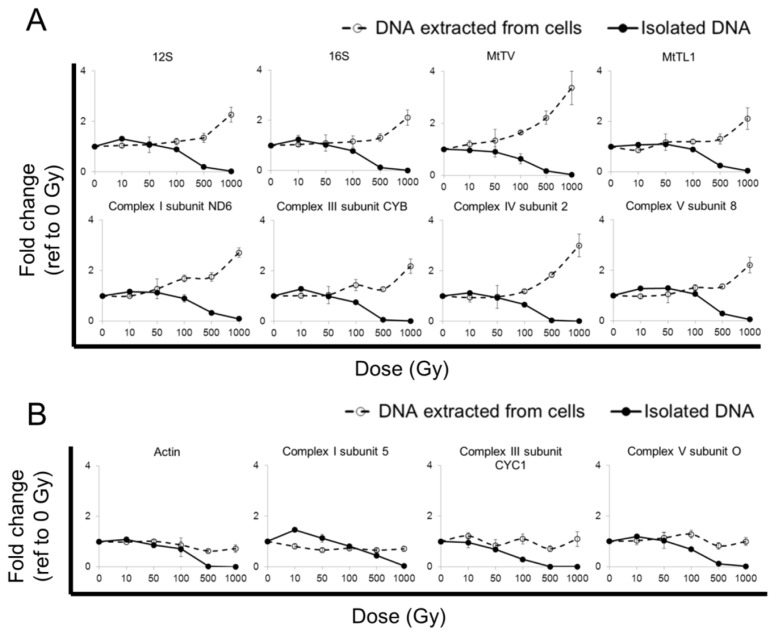

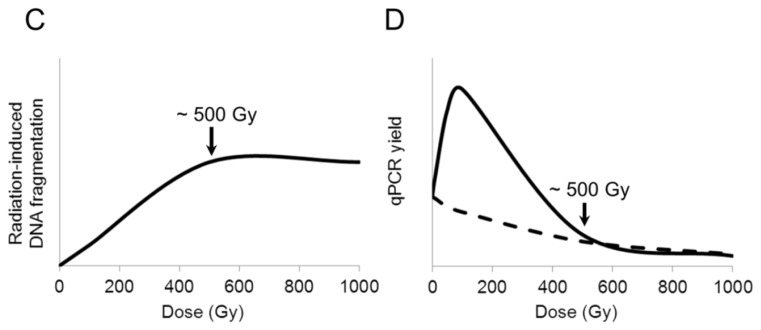
Gene copy number quantification in cells or isolated (cell-free) total DNA after gamma irradiation at 0.2–1.2 °C. JURKAT cells (dashed line) or isolated total DNA (solid line) (*N* = 3) were exposed to 0 (control), 10, 50, 100, 500 and 1000 Gy of gamma radiation at 0.2–1.2 °C. DNA samples were analysed by qPCR and quantified with reference to the un-irradiated sample: 2 ^Cq (un-irradiated sample) − Cq (irradiated sample)^. Changes in qPCR yield of the tested mitochondrial (**A**) and nuclear (**B**) genes are shown as fold changes. The data show that qPCR yield between cellular DNA and isolated DNA templates are similar after 10 to 100 Gy of radiation exposure. From 500 Gy and higher, a slight increase is noted in cellular DNA template, while a decrease is observed in isolated DNA template; (**C**) The radiation damage to nucleotides and DNA fragmentation is assumed to increase linearly with dose [27]. At about 500 Gy, a maximal level of radiation-induced DNA fragmentation is reached, as all templates have been fragmented; (**D**) The dashed line shows the theoretically expected qPCR yield from a radiation-damaged, but un-fragmented DNA template. The yield gradually decreases with higher radiation dose (likely due to increasing changes of the nucleotides that render them increasingly un-amplifiable). The solid line represents the qPCR yield from a DNA template becoming increasingly fragmented by radiation. A higher than expected level of yield is measured from samples irradiated by 10 to 100 Gy of ionizing radiation. A further dose increase, from 500 Gy onwards, leads to a reduction in qPCR yield (likely due to a near complete DNA template fragmentation; thus, genes are un-amplifiable).

In contrast to cells irradiated at room temperature, mitochondrial gene copy numbers of cells irradiated at low temperature only showed small variations (±~40%) in mitochondrial gene copy numbers across 10 to 100 Gy (Figure 3A; dashed line). A trend increase in mitochondrial gene copy numbers could be detected at 500 Gy or above (Figure 3A; dashed line), *i.e.*, the doses where marked DNA fragmentation occurred (Figure 2B, top image, 500 and 1000 Gy lanes). The nuclear gene copy numbers remained mostly unchanged (±~40%) within 10 to 100 Gy and only showed a slight trend increase with further dose increase (Figure 3B; dashed lines).

In cell-free DNA irradiated at low temperature, all tested mitochondrial (Figure 3A, solid lines) and nuclear (Figure 3B, solid lines) genes measured by qPCR showed a gradual decrease in the amount of gene detectable with increasing radiation dose, which is likely to be associated with an increase in DNA damage [16–19].

### 2.5. Gel Analysis of DNA Template Fragmentation after Radiation Exposure at Low Temperature

DNA from cells irradiated at low temperature showed a size reduction of the major band (Figure 2B, top image, arrows) and small fragment formation only at 500 and 1000 Gy (Figure 2B, top image, boxed region and profile plot).

Isolated DNA directly exposed to radiation at low temperature showed a lower level of smearing/fragment formation at all tested doses (Figure 2B; bottom image, profile plot) when compared to that at room temperature (Figure 2A; bottom image, profile plot). A reduction in qPCR yield with dose increase was observed (Figure 3A,B; solid lines).

A delayed increase in mitochondrial DNA copy numbers after radiation stimulation has previously been reported [2–9]. Our results, thus, appear consistent with earlier studies and further show that such an increase in mitochondrial DNA copy numbers is detectable by qPCR soon after irradiation (within 30 min) (Figure 1; dashed lines). However, for a number of reasons, these findings point to a methodological error in the estimation of gene copy number by qPCR.

The mitochondrial genome contains a single copy of each of its 37 mitochondrial genes [28]. If there are multiple mitochondrial DNAs, the copy number of each mitochondrial gene should multiply accordingly. An averaged quantity of a few of mitochondrial genes can thus be used to estimate the total mitochondrial DNA copy number [3]. However, we observed, for example, after 10 Gy of irradiation, a dose commonly used in radiation studies, that the MtTL1 gene copy number increased by only 10% in contrast to a 230% increase in copy number for the gene of Complex IV subunit 2 (Figure 1A; dashed lines). This observed variability in the radiation-induced changes in copy number between different mitochondrial genes, therefore, cautions against using an averaged gene copy number to estimate the total mitochondrial DNA content.

Further, we also observed an acute and non-linear increase in nuclear gene copy numbers with radiation dose. As seen with mitochondrial genes, the apparent increases in gene copy numbers varied over a broad range between the various nuclear genes. A 10 Gy irradiation led to a 200% increase in actin gene (Figure 1B; dashed line, Actin), a nuclear gene commonly used as a normalizer when quantifying mitochondrial DNA copy number [7]. In addition, after 10 Gy of irradiation, a ~50% increase was noted in the Complex III subunit CYC1 gene, while a 410% increase was seen in the Complex I subunit 5 gene (Figure 1B; dashed lines). These findings caution against the use of the actin gene or nuclear-encoded genes of the electron transport chain to normalize against mitochondrial DNA copy number quantification from samples irradiated with 10 Gy or above. More importantly, unlike in the mitochondrial genome for which rapid increases in gene copy numbers have been reported, the measured increases in nuclear genes within 30 min after irradiation is unlikely to be a true increase in nuclear DNA copy numbers.

We, therefore, repeated the cellular irradiation experiments in cell-free conditions in order to rule out fast DNA replication as a possible explanation for the observed acute increases in gene copy numbers. An increase in qPCR yield was again observed in cell-free DNA after irradiation (Figure 1; solid lines). Taken together, the findings in cell-free systems suggest that the increase in nuclear and mitochondrial gene copy numbers after irradiation, measured as qPCR yield, is an experimental artefact and should not be interpreted as evidence for polyploidization. Consequently, the qPCR measurements on DNA from cells irradiated at room temperature (Figure 1, dashed lines), too, are likely to be an artefact rather than convincing evidence for polyploidization.

Fewer double-strand breaks are observed with the reduction in irradiation temperature (from 37 to 2 °C) [29,30]. Consistent with this, we also observed a lower level of DNA fragmentation at low temperature (Figure 2). At 500 Gy or above, DNA fragments were detected in cellular DNA (Figure 2B; top image), but not in cell-free DNA (Figure 2B; bottom image). This may due to the possible involvement of enzyme-induced DNA fragmentation within cells [31,32].

Ionizing radiation triggers apoptotic pathways in living cells, and DNA is fragmented enzymatically [31,32] if not properly repaired [33]. Under cell-free conditions, such active enzymatic cleavage should not occur. Indeed, even if irradiated with 500 Gy or more, the DNA irradiated under cell-free conditions appears un-fragmented compared to the DNA from irradiated cell (Figure 2B; bottom image).

Despite the absence of obvious fragmentation in the cell-free DNA irradiated at low temperature, the qPCR yield was markedly reduced, especially at 500 Gy or above (Figure 3A,B; solid lines). This may be due to radiation-induced nucleotide changes without DNA fragmentation. In contrast, the qPCR yield of cellular DNA exposed to 500 Gy or above (Figure 3A,B; dashed lines) was increased. This may be due to DNA fragmentation and, thus, increased amplifiability of the shortened DNA templates [34] (Figure 2B; top image; 500 and 1000 Gy lanes). It is likely that fragmentation also occurs at lower radiation doses and at low temperature. Detection of this more subtle fragmentation, however, would require more sensitive techniques, such as pulse-field gel electrophoresis [35].

Radiation causes DNA damage in a linear and dose-dependent manner [27]. The damage involves changes to the DNA nucleotides or, if more severe, single or double-stranded DNA breaks [27,35] (Figure 3C). With shorter DNA templates, qPCR primers can access the target gene sequences more readily. This increases the amplifiability of DNA [34], which can then translate into a greater than expected qPCR yield. Thus, radiation-induced DNA fragmentation may lead to apparent increases in gene copy numbers. In our study, this phenomenon was observed between 10 to 100 Gy, the dose range used in many other clinical and experimental settings. At yet higher radiation doses (500 Gy or above), qPCR yield reduces again, most likely due to the severe destruction of the DNA template, e.g., severe nucleotide damage and/or complete template fragmentation, making the target genes increasingly un-amplifiable (Figure 3D).

## 3. Experimental Section

### 3.1. Sample Preparation

Wild-type JURKAT cells were maintained at 37 °C with 5% CO_2_ in DMEM supplemented with 10% FBS, 2 mM l-glutamine, 100 units/mL penicillin and 100 μg/mL streptomycin until the day of the experiment. Cells were irradiated at a density of 0.16 × 10^6^ in 1 mL medium (described above) in a 1.5 mL-Eppendorf tube at room temperature or on ice (see *Gamma Irradiation*).

In addition, isolated DNA extracted from the non-irradiated cells was diluted to 1 ng/μL using DEPC-treated water. One millilitre of the diluted DNA in a 1.5 mL-Eppendorf tube was irradiated at room temperature or on ice (see Section 3.2.).

### 3.2. Gamma Irradiation

Four irradiation experiments were performed: (i) cells irradiated at room temperature; (ii) cells irradiated on ice; (iii) isolated DNA irradiated at room temperature; and (iv) isolated DNA irradiated on ice. For experiment (ii) and (iv), samples were kept on ice for 20 min before the irradiation, which was performed in a chamber of crushed ice with temperatures ranging from 0.2 to 1.2 °C during the exposure. The controls were kept at room temperature (21 ± 2 °C at the time of this experiment) or on ice (for experiments (ii) and (iv)) and were not irradiated.

Cells or isolated DNA were gamma irradiated at 10, 50, 100, 500 or 1000 Gy using a ^60^Co irradiator (GammaCell 220). This wide dose range was chosen to cover the doses applied in clinical [36–38] and experimental settings [3,29,39–45], especially some other radiation studies, e.g., 150 Gy used in [46], 250 Gy used in [35] and 560 Gy used in [47].

The dose rate of 39.0 ± 0.8 Gy/min was determined using the standard Fricke dosimeter. At this dose rate, the effect of the dose during transit of the GammaCell 220 chamber (5.2 ± 0.4 Gy) was significant and was taken into account when calculating the exposure times.

### 3.3. DNA Isolation and Gel Electrophoresis

DNA extraction was performed 25 min after the start of the each irradiation. DNA extraction was performed by the PureLink™ Genomic DNA Kit (Invitrogen, Carlsbad, CA, USA) following the manufacturer’s protocol. To remove residual contaminant, RNase A and Proteinase K treatments were done for DNA samples. DNA was eluted in 25 μL of diethylpyrocarbonate (DEPC)-treated water. The concentration of the DNA was determined using the NanoDrop 2000c Spectrophotometer (Thermo Fisher Scientific, Waltham, MA, USA). The purity of the extracted DNA was assessed spectrophotometrically using the A260/A280 ratio. The quality of the extracted DNA samples was assessed by 1.5% agarose gel electrophoresis. DNA was further diluted to 1 ng/μL for the subsequent qPCR assay.

### 3.4. qPCR

PCR primers were selected from published literature (Table 1). Primer specificity was confirmed in-house by melt curve analysis prior to the experiment. The specificity of the Complex IV subunit 2 primer set was further confirmed by sequencing (Accession: AF004339), as the original paper did not specify the target subunit [48].

qPCR was performed using the CFX 384™ Real-Time PCR Detection System (BioRad, Hercules, CA, USA). Diluted DNA of 1 μL was added to 4 μL of reaction mixture containing 2.5 μL of SsoFast™ EvaGreen^®^ Supermix (BioRad, Hercules, CA, USA) and 5 pM of each of the forward and reverse primers. Each sample was run in duplicate. Annealing temperature was determined empirically to accommodate all primers in a single qPCR run. The thermal cycling conditions were 98 °C for 30 s, followed by 45 cycles at 98 °C for 5 s and 63 °C for 10 s. The amplification efficiencies of our targets [49] using this PCR condition were within the range of ~90% to 110% (Supplementary Information).

Twelve different PCR primer sets were used in this study, and they may have differences in their ability to hybridize to and initiate amplification from the template. Thus, a few more cycles (5 cycles more than the routine practice (40 cycles)) are needed to ensure that the reaction of all 12 tested genes has sufficiently reached the detection threshold (Cq value, please see below). At the end of the 45th cycle, the temperature was raised to 72 °C for 10 min to ensure the complete extension of the products. A melt curve analysis was performed after the qPCR to confirm the specificity of the results.

The mean Cq (quantification cycle) value of each sample was quantified using the CFX Manager™ Software (version 1.5) (BioRad, Hercules, CA, USA), and the Cq value is inversely proportional to the starting amount of DNA template, which is the basis for estimating the gene copy number [6]. qPCR data analysis was performed using the ΔCq method referencing to the un-irradiated sample, *i.e.*, 2 ^Cq (un-irradiated sample) − Cq (irradiated sample)^[14,15]. This quantification method compares the same gene between the un-irradiated and irradiated samples. A relative fold change >1 indicated an increase in gene copy number after irradiation, while a value <1 represented the opposite. In this study, fold changes of ≥2, *i.e.*, more than a 100% increase relative to the un-irradiated control, are regarded as substantial changes in gene copy numbers.

## 4. Conclusions

In summary, our results show that estimation errors are introduced when using qPCR to quantify DNA copy numbers from potentially fragmented DNA template. DNA fragmentation in this study was induced by ionizing radiation, but the findings may be extrapolated to other means of damage that results in DNA fragment formation. Any apparent polyploidization, as detected by qPCR, should be cross-validated, e.g., by flow cytometry [56] or fluorescence microscopy [1,56].

## Supplementary Information



## Figures and Tables

**Table 1:** PCR primers. The listed primers were used to examine the expression of the following mitochondrial genes—ribosomal RNA: 12S, 16S; transfer RNA; MtTL1 (mitochondrially encoded tRNA leucine 1 (UUA/G)), MtTV (mitochondrially encoded tRNA(Val)); messenger RNA: Complex I subunit ND6; Complex III subunit CYB; Complex IV subunit 2; Complex V subunit 8. The following nuclear genes were also investigated: actin, Complex I subunit 5, Complex III subunit CYC1 and Complex V subunit O.

Coding origin	Gene name	Forward primer	Reverse primer	Reference
Mitochondria	12S	CCCAAACTGGGATTAGATACCC	GTTTGCTGAAGATGGCGGTA	[50]
	16S	GCCTGTTTACCAAAAACATCAC	CTCCATAGGGTCTTCTCGTCTT	[50]
	MTTV	CTGGAAAGTGCACTTGGACG	GGGTAAATGGTTTGGCTAAGG	[51]
	MTTL1	TATACCCACACCCACCCAAG	GCGATTAGAATGGGTACAAT	[51]
	Complex I subunit ND6	GGATCCTCCCGAATCAAC	GTAGGATTGGTGCTGTGG	[52]
	Complex III subunit CYB	TGAAACTTCGGCTCACTCCT	AATGTATGGGATGGCGGATA	[53]
	Complex IV subunit 2	CAGGAAATAGAAACCGTCTGAACTATCCTG	CTGTGGTTTGCTCCACAGATTTCAGTGCAT	[48]
	Complex V subunit 8	ATGGCCCACCATAATTACCC	GCAATGAATGAAGCGAACAG	[54]

Nucleus	Actin	GTGGGGCGCCCCAGGCACCA	CTCCTTAATGTCACGCACGATTTC	[48]
	Complex I subunit 5	GAGAAGCTGGCTATGGTTAAAGCG	CCACTAATGGCTCCCATAGTTTCC	[52]
	Complex III subunit CYC1	CCAAAACCATACCCCAACAG	TATGCCAGCTTCCGACTCTT	[53]
	Complex V subunit O	ACCCAAGGAGTCGTTTCTGC	TTAGACAATCTCCCGCATAGC	[55]
