# Supplementary material for: Apparent Polyploidization after Gamma Irradiation: Pitfalls in the Use of Quantitative Polymerase Chain Reaction (qPCR) for the Estimation of Mitochondrial and Nuclear DNA Gene Copy Numbers
Source: Int J Mol Sci. 2013 May 30;14(6):11544–59. doi: 10.3390/ijms140611544 (PMC3709747; doi:10.3390/ijms140611544)
Supplement: Supplementary file 1 [file ijms-14-11544-s001.pdf]

Supplementary Information

Figure S1. The PCR efficiency was determined by serial dilution of cDNA template of known concentration. The efficiency (E) of each primer set to amplify the target gene is shown in each graph.

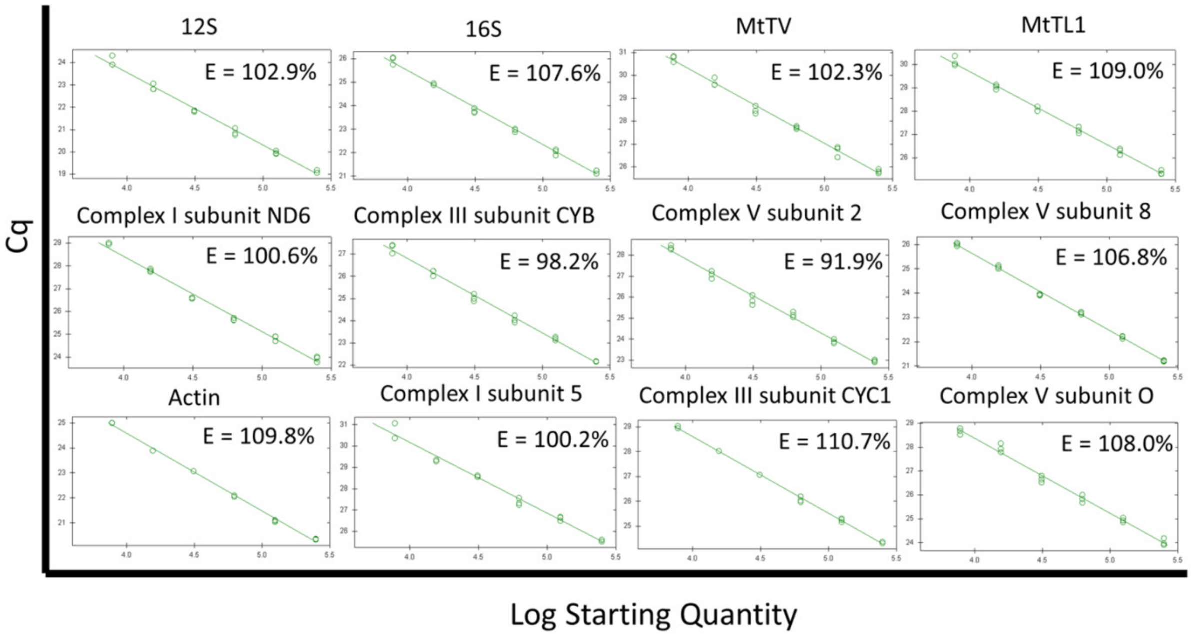

© 2013 by the authors; licensee MDPI, Basel, Switzerland. This article is an open access article distributed under the terms and conditions of the Creative Commons Attribution license (http://creativecommons.org/licenses/by/3.0/).
